# Crystal structure of (6-bromo-2-oxo-2*H*-chromen-4-yl)methyl morpholine-4-carbodi­thio­ate

**DOI:** 10.1107/S2056989015011007

**Published:** 2015-06-13

**Authors:** K. Mahesh Kumar, K. R. Roopashree, M. Vinduvahini, O. Kotresh, H. C. Devarajegowda

**Affiliations:** a Department of Chemistry, Karnatak University’s Karnatak Science College, Dharwad, Karnataka 580 001, India; b Department of Physics, Yuvaraja’s College (Constituent College), University of Mysore, Mysore 570 005, Karnataka, India; c Department of Physics, Sri D. Devaraja Urs Govt. First Grade College, Hunsur 571 105, Mysore District, Karnataka, India

**Keywords:** crystal structure, coumarins, di­thio­carbamates, biological applications, hydrogen bonding, π–π inter­actions

## Abstract

In the title compound, C_15_H_14_BrNO_3_S_2_, the 2*H*-chromene ring system is nearly planar, with a maximum deviation of 0.034 (2) Å, and the morpholine ring adopts a chair conformation. The dihedral angle between best plane through the 2*H*-chromene ring system and the morpholine ring is 86.32 (9)°. Intra­molecular C—H⋯S hydrogen bonds are observed. In the crystal, inversion-related C—H⋯S and C—H⋯O inter­actions generate *R*_2_^2^(10) and *R*_2_^2^(8) rings patterns, respectively. In addition, the crystal packing features π–π inter­actions between fused benzene rings [centroid–centroid distance = 3.7558 (12) Å].

## Related literature   

For biological applications of coumarins and di­thio­carbamates, see: D’hooghe & De Kimpe (2006 ▸); Hesse & Kirsch (2002 ▸); Jung *et al.* (2001 ▸, 2004 ▸); Lee *et al.* (1998 ▸); Melagraki *et al.* (2009 ▸); Schönenberger & Lippert (1972 ▸). For standard bond lengths, see: Devarajegowda *et al.* (2013 ▸). For a related structure and the synthesis of the title compound, see: Devarajegowda *et al.* (2013 ▸).
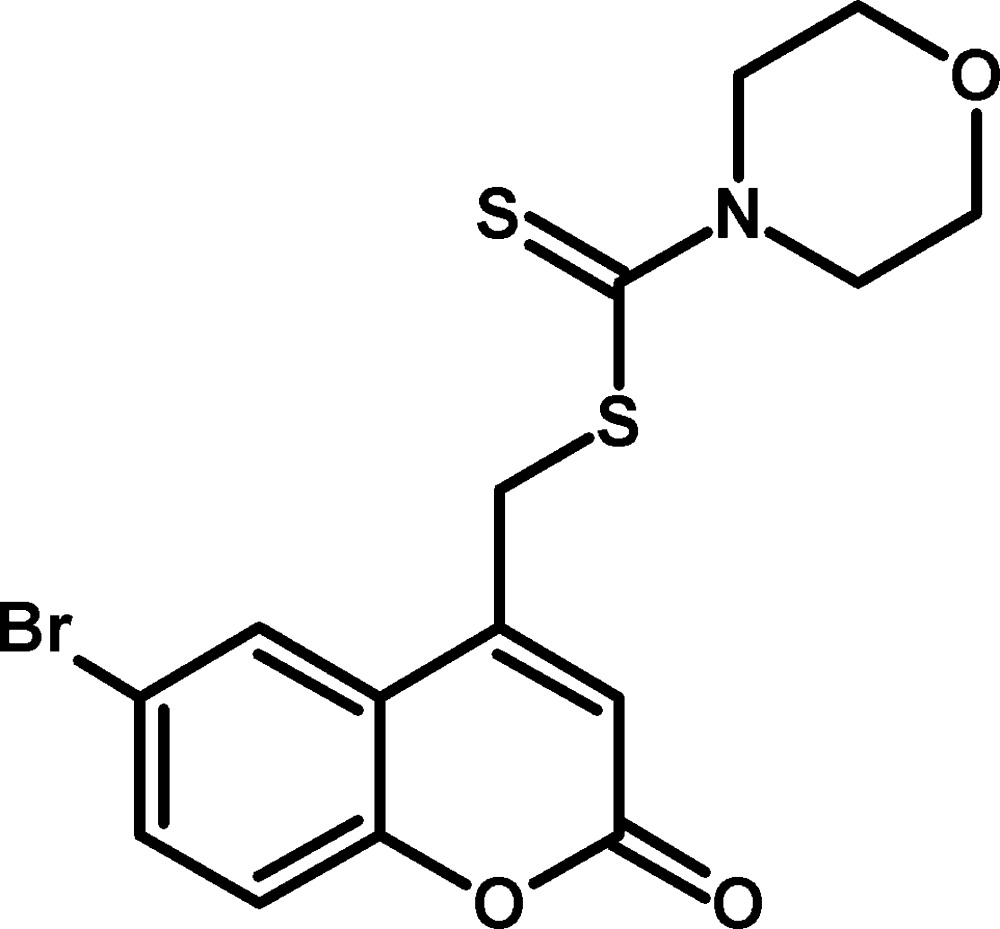


## Experimental   

### Crystal data  

C_15_H_14_BrNO_3_S_2_
*M* *_r_* = 400.30
Triclinic, 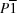

*a* = 7.0500 (3) Å
*b* = 7.6049 (3) Å
*c* = 15.1376 (7) Å
α = 78.782 (2)°
β = 88.549 (2)°
γ = 78.515 (2)°
*V* = 780.07 (6) Å^3^
*Z* = 2
Mo *K*α radiation
μ = 2.91 mm^−1^
*T* = 296 K
0.24 × 0.20 × 0.12 mm

### Data collection  

Bruker SMART CCD area-detector diffractometer
Absorption correction: ψ scan (*SADABS*; Sheldrick, 2007 ▸) *T*_min_ = 0.770, *T*_max_ = 1.000
13789 measured reflections
3224 independent reflections
2806 reflections with *I* > 2σ(*I*)
*R*_int_ = 0.027

### Refinement  

*R*[*F* ^2^ > 2σ(*F* ^2^)] = 0.026
*wR*(*F* ^2^) = 0.065
*S* = 1.03
3224 reflections
199 parameters
H-atom parameters constrained
Δρ_max_ = 0.26 e Å^−3^
Δρ_min_ = −0.43 e Å^−3^

### 

Data collection: *SMART* (Bruker, 2001 ▸); cell refinement: *SAINT* (Bruker, 2001 ▸); data reduction: *SAINT*; program(s) used to solve structure: *SHELXS97* (Sheldrick, 2008 ▸); program(s) used to refine structure: *SHELXL2014* (Sheldrick, 2015 ▸); molecular graphics: *ORTEP-3 for Windows* (Farrugia, 2012 ▸); software used to prepare material for publication: *SHELXL2014*.

## Supplementary Material

Crystal structure: contains datablock(s) I, global. DOI: 10.1107/S2056989015011007/bq2399sup1.cif

Structure factors: contains datablock(s) I. DOI: 10.1107/S2056989015011007/bq2399Isup2.hkl

Supporting information file. DOI: 10.1107/S2056989015011007/bq2399Isup3.cml

**Fig. 1.**
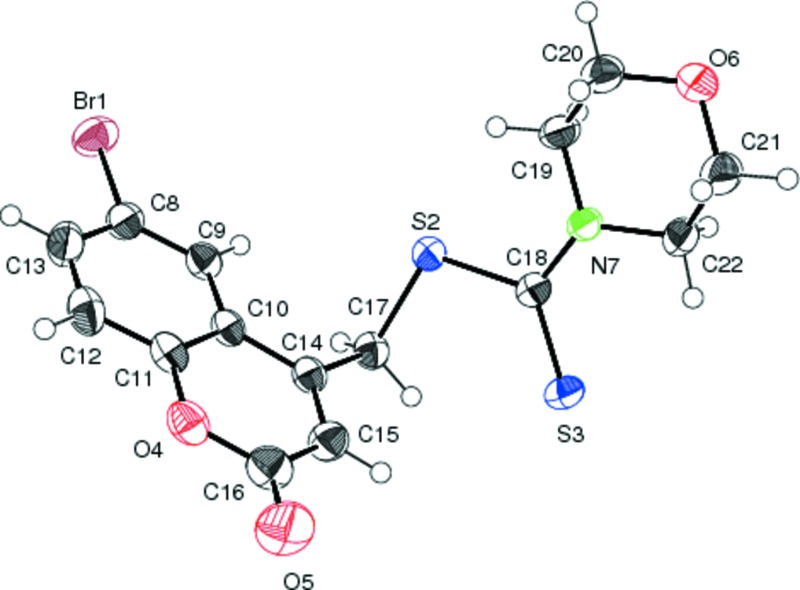
The molecular structure of the title compound. Displacement ellipsoids are drawn at the 50% probability level. Hydrogen atoms are shown as spheres of arbitrary radius.

. DOI: 10.1107/S2056989015011007/bq2399fig1.tif
The mol­ecular structure of the title compound. Displacement ellipsoids are drawn at the 50% probability level. Hydrogen atoms are shown as spheres of arbitrary radius.

**Fig. 2.**
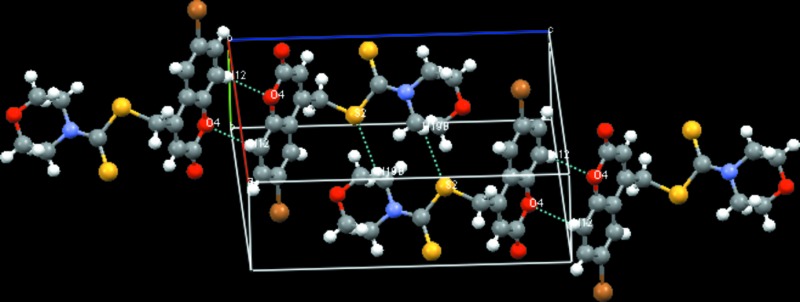
Crystal packing for the title compound with hydrogen bonds drawn as dashed lines.

. DOI: 10.1107/S2056989015011007/bq2399fig2.tif
Crystal packing for the title compound with hydrogen bonds drawn as dashed lines.

CCDC reference: 1405247

Additional supporting information:  crystallographic information; 3D view; checkCIF report

## Figures and Tables

**Table 1:** Hydrogen-bond geometry (, )

*D*H*A*	*D*H	H*A*	*D* *A*	*D*H*A*
C17H17*A*O5^i^	0.97	2.53	3.501(2)	176
C17H17*B*S3	0.97	2.55	3.1633(16)	121
C19H19*A*S2	0.97	2.37	2.864(2)	111
C22H22*B*S3	0.97	2.61	3.0486(19)	108

Symmetry code: (i) 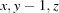.

## supplementary crystallographic information

### S1. Comment

In recent years, much attention has been directed towards the synthesis of substituted coumarins owing to their tremendous application in various research fields including biological science and medicinal chemistry. Substituted coumarin derivatives are components of numerous natural products like warfarin, phenprocoumon, coumatetralyl, carbochromen, bromadialone, *etc*. These compounds also exhibit a wide band of biological activities including antibacterial, anti-HIV (Hesse & Kirsch, 2002), antiviral (Lee *et al.*, 1998), anticoagulant (Jung *et al.*, 2001), antioxidant (Melagraki *et al.*, 2009) and anticancer activities (Jung *et al.*,(2004). Carbon–sulfur bond formation is a fundamental approach to bring sulfur into organic compounds, and this has received much attention due to its occurrence in many molecules that are of biological and pharmaceutical importance. The antibacterial and antifungal activities of dithiocarbamates were reported to arise by the reaction with HS-groups of the physiologically important enzymes by transferring the alkyl group of the dithioester to the HS-function of the enzyme (Schönenberger & Lippert, 1972). Organic dithiocarbamates, ubiquitously found in a variety of biologically active molecules (Dhooghe & De Kimpe, 2006), are of high importance in academia as well as in industry.

In view of the various physiological activities of coumarins and dithiocarbamates, our current studies are focused on the development of new routes for the synthesis of coumarins incorporating dithiocarbamate moieties.

The asymmetric unit of (6-bromo-2-oxo-2*H*-chromen-4-yl)methyl morpholine-4-carbodi thioate is shown in Fig. 1. The 2*H*-chromene ring systems is nearly planar, with a maximum deviation of 0.0337 (23) Å for the atom C16 and the morpholine ring adopts a chair conformation. The dihedral angle between the 2*H*-chromene ring and the morpholine ring is 86.32 (9) °. In the crystal structure, intermolecular C—H···O and intramolecular C–H···S hydrogen bonds are observed (Table 1) and inversion related C—H···S and C—H···O interactions generate *R*_2_^2^(10) and *R*_2_^2^(8) rings pattern respectively. In addition, the crystal packing is stabilized by π–π [C_g_ (3)– C_g_(3);C8–C13] interactions between fused benzene rings [centroid- centroid distance = 3.7558 (12)].

### S2. Experimental

All the chemicals used were of analytical reagent grade and were used directly without further purification. The title compound was synthesized according to the reported method (Devarajegowda *et al.*, 2013). The compound is recrystallized by ethanol-chloroform mixture. Colourless needles of the title compound were grown from a mixed solution of Ethanol/Chloroform (V/V = 2/1) by slow evaporation at room temperature. Yield =72%, m.p.: 433–435 K

### S3. Refinement

All H atoms were positioned geometrically, with C—H = 0.93 Å for aromatic H and C—H = 0.97 Å for methylene H and refined using a riding model with *U*_iso_(H) = 1.2*U*_eq_(C) for aromatic and methylene H.

### Crystal data

**Table:**

C_15_H_14_BrNO_3_S_2_	*F*(000) = 404
*M**_r_* = 400.30	*D*_x_ = 1.704 Mg m^−^^3^
Triclinic, *P*1	Melting point: 435 K
*a* = 7.0500 (3) Å	Mo *K*α radiation, λ = 0.71073 Å
*b* = 7.6049 (3) Å	Cell parameters from 3224 reflections
*c* = 15.1376 (7) Å	θ = 2.7–26.5°
α = 78.782 (2)°	µ = 2.91 mm^−^^1^
β = 88.549 (2)°	*T* = 296 K
γ = 78.515 (2)°	Plate, colourless
*V* = 780.07 (6) Å^3^	0.24 × 0.20 × 0.12 mm
*Z* = 2	

### Data collection

**Table:**

Bruker SMART CCD area-detector diffractometer	3224 independent reflections
Radiation source: fine-focus sealed tube	2806 reflections with *I* > 2σ(*I*)
Graphite monochromator	*R*_int_ = 0.027
ω and φ scans	θ_max_ = 26.5°, θ_min_ = 2.7°
Absorption correction: ψ scan (*SADABS*; Sheldrick, 2007)	*h* = −8→8
*T*_min_ = 0.770, *T*_max_ = 1.000	*k* = −9→9
13789 measured reflections	*l* = −18→18

### Refinement

**Table:**

Refinement on *F*^2^	Primary atom site location: structure-invariant direct methods
Least-squares matrix: full	Secondary atom site location: difference Fourier map
*R*[*F*^2^ > 2σ(*F*^2^)] = 0.026	Hydrogen site location: inferred from neighbouring sites
*wR*(*F*^2^) = 0.065	H-atom parameters constrained
*S* = 1.03	*w* = 1/[σ^2^(*F*_o_^2^) + (0.0328*P*)^2^ + 0.2957*P*] where *P* = (*F*_o_^2^ + 2*F*_c_^2^)/3
3224 reflections	(Δ/σ)_max_ = 0.001
199 parameters	Δρ_max_ = 0.26 e Å^−^^3^
0 restraints	Δρ_min_ = −0.43 e Å^−^^3^

### Special details

**Table:**

Geometry. All e.s.d.'s (except the e.s.d. in the dihedral angle between two l.s. planes) are estimated using the full covariance matrix. The cell e.s.d.'s are taken into account individually in the estimation of e.s.d.'s in distances, angles and torsion angles; correlations between e.s.d.'s in cell parameters are only used when they are defined by crystal symmetry. An approximate (isotropic) treatment of cell e.s.d.'s is used for estimating e.s.d.'s involving l.s. planes.

### Fractional atomic coordinates and isotropic or equivalent isotropic displacement parameters (Å^2^)

**Table:**

	*x*	*y*	*z*	*U*_iso_*/*U*_eq_	
Br1	0.19477 (3)	0.33461 (3)	0.89681 (2)	0.05002 (9)	
S2	0.70559 (7)	0.58659 (7)	0.62666 (3)	0.03418 (12)	
S3	1.06683 (7)	0.72703 (8)	0.56091 (3)	0.03905 (13)	
O6	0.5420 (2)	0.9077 (2)	0.29823 (10)	0.0520 (4)	
O4	0.6263 (2)	0.94245 (19)	0.89038 (9)	0.0404 (3)	
O5	0.8534 (3)	1.0955 (2)	0.84135 (14)	0.0650 (5)	
N7	0.7479 (2)	0.7625 (2)	0.46382 (10)	0.0303 (3)	
C8	0.3315 (3)	0.5266 (3)	0.89259 (13)	0.0373 (4)	
C9	0.4939 (3)	0.5289 (3)	0.84039 (12)	0.0337 (4)	
H9	0.5346	0.4379	0.8070	0.040*	
C10	0.5976 (3)	0.6687 (2)	0.83777 (11)	0.0299 (4)	
C11	0.5313 (3)	0.8021 (3)	0.88881 (12)	0.0345 (4)	
C12	0.3686 (3)	0.7987 (3)	0.94128 (14)	0.0441 (5)	
H12	0.3272	0.8889	0.9750	0.053*	
C13	0.2683 (3)	0.6601 (3)	0.94310 (14)	0.0450 (5)	
H13	0.1585	0.6561	0.9782	0.054*	
C14	0.7717 (3)	0.6820 (2)	0.78610 (11)	0.0302 (4)	
C15	0.8595 (3)	0.8222 (3)	0.78884 (14)	0.0381 (4)	
H15	0.9729	0.8289	0.7567	0.046*	
C16	0.7858 (3)	0.9630 (3)	0.83943 (15)	0.0428 (5)	
C17	0.8495 (3)	0.5444 (3)	0.72867 (12)	0.0328 (4)	
H17A	0.8481	0.4223	0.7622	0.039*	
H17B	0.9826	0.5515	0.7133	0.039*	
C18	0.8436 (2)	0.7021 (2)	0.54254 (12)	0.0275 (4)	
C19	0.5618 (3)	0.7172 (3)	0.44561 (14)	0.0392 (5)	
H19A	0.4913	0.6973	0.5014	0.047*	
H19B	0.5848	0.6047	0.4223	0.047*	
C20	0.4422 (3)	0.8674 (3)	0.37879 (14)	0.0418 (5)	
H20A	0.3232	0.8309	0.3659	0.050*	
H20B	0.4081	0.9764	0.4047	0.050*	
C21	0.7118 (3)	0.9691 (4)	0.31608 (16)	0.0503 (6)	
H21A	0.6753	1.0799	0.3403	0.060*	
H21B	0.7785	0.9988	0.2600	0.060*	
C22	0.8474 (3)	0.8295 (3)	0.38134 (13)	0.0381 (4)	
H22A	0.9027	0.7273	0.3530	0.046*	
H22B	0.9525	0.8835	0.3969	0.046*	

### Atomic displacement parameters (Å^2^)

**Table:**

	*U*^11^	*U*^22^	*U*^33^	*U*^12^	*U*^13^	*U*^23^
Br1	0.04201 (14)	0.05131 (15)	0.05488 (15)	−0.01752 (10)	−0.00222 (10)	0.00309 (10)
S2	0.0345 (2)	0.0421 (3)	0.0294 (2)	−0.0157 (2)	0.00236 (18)	−0.00731 (19)
S3	0.0262 (2)	0.0502 (3)	0.0414 (3)	−0.0118 (2)	−0.00145 (19)	−0.0059 (2)
O6	0.0422 (8)	0.0743 (11)	0.0365 (8)	−0.0190 (8)	−0.0082 (6)	0.0050 (7)
O4	0.0543 (9)	0.0348 (7)	0.0348 (7)	−0.0096 (6)	−0.0005 (6)	−0.0120 (6)
O5	0.0792 (13)	0.0475 (10)	0.0813 (13)	−0.0312 (9)	0.0080 (10)	−0.0262 (9)
N7	0.0259 (7)	0.0327 (8)	0.0322 (8)	−0.0083 (6)	0.0005 (6)	−0.0036 (6)
C8	0.0350 (10)	0.0413 (11)	0.0321 (10)	−0.0085 (9)	−0.0031 (8)	0.0025 (8)
C9	0.0386 (10)	0.0339 (10)	0.0278 (9)	−0.0064 (8)	−0.0018 (8)	−0.0047 (7)
C10	0.0356 (9)	0.0293 (9)	0.0225 (8)	−0.0036 (7)	−0.0026 (7)	−0.0022 (7)
C11	0.0438 (11)	0.0321 (10)	0.0260 (9)	−0.0038 (8)	−0.0029 (8)	−0.0052 (7)
C12	0.0507 (12)	0.0463 (12)	0.0335 (10)	−0.0010 (10)	0.0067 (9)	−0.0133 (9)
C13	0.0392 (11)	0.0562 (14)	0.0358 (11)	−0.0049 (10)	0.0067 (9)	−0.0052 (10)
C14	0.0330 (9)	0.0296 (9)	0.0256 (8)	−0.0035 (7)	−0.0032 (7)	−0.0018 (7)
C15	0.0382 (10)	0.0375 (11)	0.0395 (10)	−0.0099 (8)	0.0014 (8)	−0.0073 (8)
C16	0.0522 (13)	0.0352 (11)	0.0422 (11)	−0.0112 (9)	−0.0040 (10)	−0.0072 (9)
C17	0.0335 (10)	0.0327 (10)	0.0304 (9)	−0.0033 (8)	0.0009 (7)	−0.0054 (8)
C18	0.0269 (9)	0.0238 (8)	0.0325 (9)	−0.0037 (7)	0.0026 (7)	−0.0089 (7)
C19	0.0309 (10)	0.0447 (12)	0.0419 (11)	−0.0153 (9)	−0.0047 (8)	0.0005 (9)
C20	0.0305 (10)	0.0471 (12)	0.0449 (11)	−0.0067 (9)	−0.0030 (8)	−0.0022 (9)
C21	0.0423 (12)	0.0611 (15)	0.0435 (12)	−0.0203 (11)	−0.0036 (9)	0.0103 (11)
C22	0.0293 (9)	0.0480 (12)	0.0356 (10)	−0.0085 (9)	0.0050 (8)	−0.0044 (9)

### Geometric parameters (Å, º)

**Table:**

Br1—C8	1.894 (2)	C12—C13	1.377 (3)
S2—C18	1.7839 (18)	C12—H12	0.9300
S2—C17	1.8095 (19)	C13—H13	0.9300
S3—C18	1.6585 (18)	C14—C15	1.343 (3)
O6—C20	1.405 (3)	C14—C17	1.501 (3)
O6—C21	1.418 (3)	C15—C16	1.442 (3)
O4—C16	1.365 (3)	C15—H15	0.9300
O4—C11	1.373 (2)	C17—H17A	0.9700
O5—C16	1.202 (3)	C17—H17B	0.9700
N7—C18	1.338 (2)	C19—C20	1.500 (3)
N7—C19	1.466 (2)	C19—H19A	0.9700
N7—C22	1.470 (2)	C19—H19B	0.9700
C8—C9	1.376 (3)	C20—H20A	0.9700
C8—C13	1.385 (3)	C20—H20B	0.9700
C9—C10	1.400 (3)	C21—C22	1.501 (3)
C9—H9	0.9300	C21—H21A	0.9700
C10—C11	1.394 (3)	C21—H21B	0.9700
C10—C14	1.448 (3)	C22—H22A	0.9700
C11—C12	1.380 (3)	C22—H22B	0.9700
			
C18—S2—C17	104.28 (9)	C14—C17—S2	110.63 (13)
C20—O6—C21	109.57 (16)	C14—C17—H17A	109.5
C16—O4—C11	121.90 (15)	S2—C17—H17A	109.5
C18—N7—C19	123.45 (15)	C14—C17—H17B	109.5
C18—N7—C22	121.02 (15)	S2—C17—H17B	109.5
C19—N7—C22	112.94 (15)	H17A—C17—H17B	108.1
C9—C8—C13	121.3 (2)	N7—C18—S3	124.57 (14)
C9—C8—Br1	119.16 (16)	N7—C18—S2	112.39 (13)
C13—C8—Br1	119.54 (16)	S3—C18—S2	123.03 (11)
C8—C9—C10	119.64 (18)	N7—C19—C20	111.25 (16)
C8—C9—H9	120.2	N7—C19—H19A	109.4
C10—C9—H9	120.2	C20—C19—H19A	109.4
C11—C10—C9	118.19 (18)	N7—C19—H19B	109.4
C11—C10—C14	117.86 (17)	C20—C19—H19B	109.4
C9—C10—C14	123.94 (17)	H19A—C19—H19B	108.0
O4—C11—C12	116.55 (18)	O6—C20—C19	111.60 (17)
O4—C11—C10	121.57 (18)	O6—C20—H20A	109.3
C12—C11—C10	121.87 (19)	C19—C20—H20A	109.3
C13—C12—C11	119.2 (2)	O6—C20—H20B	109.3
C13—C12—H12	120.4	C19—C20—H20B	109.3
C11—C12—H12	120.4	H20A—C20—H20B	108.0
C12—C13—C8	119.8 (2)	O6—C21—C22	112.72 (18)
C12—C13—H13	120.1	O6—C21—H21A	109.0
C8—C13—H13	120.1	C22—C21—H21A	109.0
C15—C14—C10	118.75 (17)	O6—C21—H21B	109.0
C15—C14—C17	120.61 (18)	C22—C21—H21B	109.0
C10—C14—C17	120.62 (16)	H21A—C21—H21B	107.8
C14—C15—C16	122.92 (19)	N7—C22—C21	111.57 (16)
C14—C15—H15	118.5	N7—C22—H22A	109.3
C16—C15—H15	118.5	C21—C22—H22A	109.3
O5—C16—O4	117.3 (2)	N7—C22—H22B	109.3
O5—C16—C15	125.9 (2)	C21—C22—H22B	109.3
O4—C16—C15	116.84 (18)	H22A—C22—H22B	108.0
			
C13—C8—C9—C10	−0.2 (3)	C11—O4—C16—O5	176.05 (19)
Br1—C8—C9—C10	−179.11 (13)	C11—O4—C16—C15	−4.6 (3)
C8—C9—C10—C11	0.0 (3)	C14—C15—C16—O5	−176.7 (2)
C8—C9—C10—C14	179.09 (17)	C14—C15—C16—O4	4.1 (3)
C16—O4—C11—C12	−178.20 (18)	C15—C14—C17—S2	−102.79 (18)
C16—O4—C11—C10	2.7 (3)	C10—C14—C17—S2	75.62 (18)
C9—C10—C11—O4	179.34 (16)	C18—S2—C17—C14	100.78 (14)
C14—C10—C11—O4	0.2 (3)	C19—N7—C18—S3	171.30 (15)
C9—C10—C11—C12	0.3 (3)	C22—N7—C18—S3	10.9 (3)
C14—C10—C11—C12	−178.90 (18)	C19—N7—C18—S2	−7.7 (2)
O4—C11—C12—C13	−179.37 (18)	C22—N7—C18—S2	−168.15 (14)
C10—C11—C12—C13	−0.3 (3)	C17—S2—C18—N7	−173.01 (13)
C11—C12—C13—C8	0.0 (3)	C17—S2—C18—S3	7.95 (14)
C9—C8—C13—C12	0.2 (3)	C18—N7—C19—C20	150.27 (18)
Br1—C8—C13—C12	179.12 (16)	C22—N7—C19—C20	−47.9 (2)
C11—C10—C14—C15	−0.7 (3)	C21—O6—C20—C19	−61.6 (2)
C9—C10—C14—C15	−179.84 (17)	N7—C19—C20—O6	56.1 (2)
C11—C10—C14—C17	−179.15 (16)	C20—O6—C21—C22	59.8 (3)
C9—C10—C14—C17	1.7 (3)	C18—N7—C22—C21	−151.82 (19)
C10—C14—C15—C16	−1.5 (3)	C19—N7—C22—C21	45.8 (2)
C17—C14—C15—C16	176.98 (18)	O6—C21—C22—N7	−51.9 (3)

### Hydrogen-bond geometry (Å, º)

**Table:**

*D*—H···*A*	*D*—H	H···*A*	*D*···*A*	*D*—H···*A*
C17—H17*A*···O5^i^	0.97	2.53	3.501 (2)	176
C17—H17*B*···S3	0.97	2.55	3.1633 (16)	121
C19—H19*A*···S2	0.97	2.37	2.864 (2)	111
C22—H22*B*···S3	0.97	2.61	3.0486 (19)	108

Symmetry code: (i) *x*, *y*−1, *z*.
