# Supplementary figures and images for: Crystal structure of (6-bromo-2-oxo-2H-chromen-4-yl)methyl morpholine-4-carbodi­thio­ate
Source: Acta Crystallogr E Crystallogr Commun. 2015 Jun 13;71(Pt 7):o489–90. doi: 10.1107/S2056989015011007 (PMC4518962; doi:10.1107/S2056989015011007)

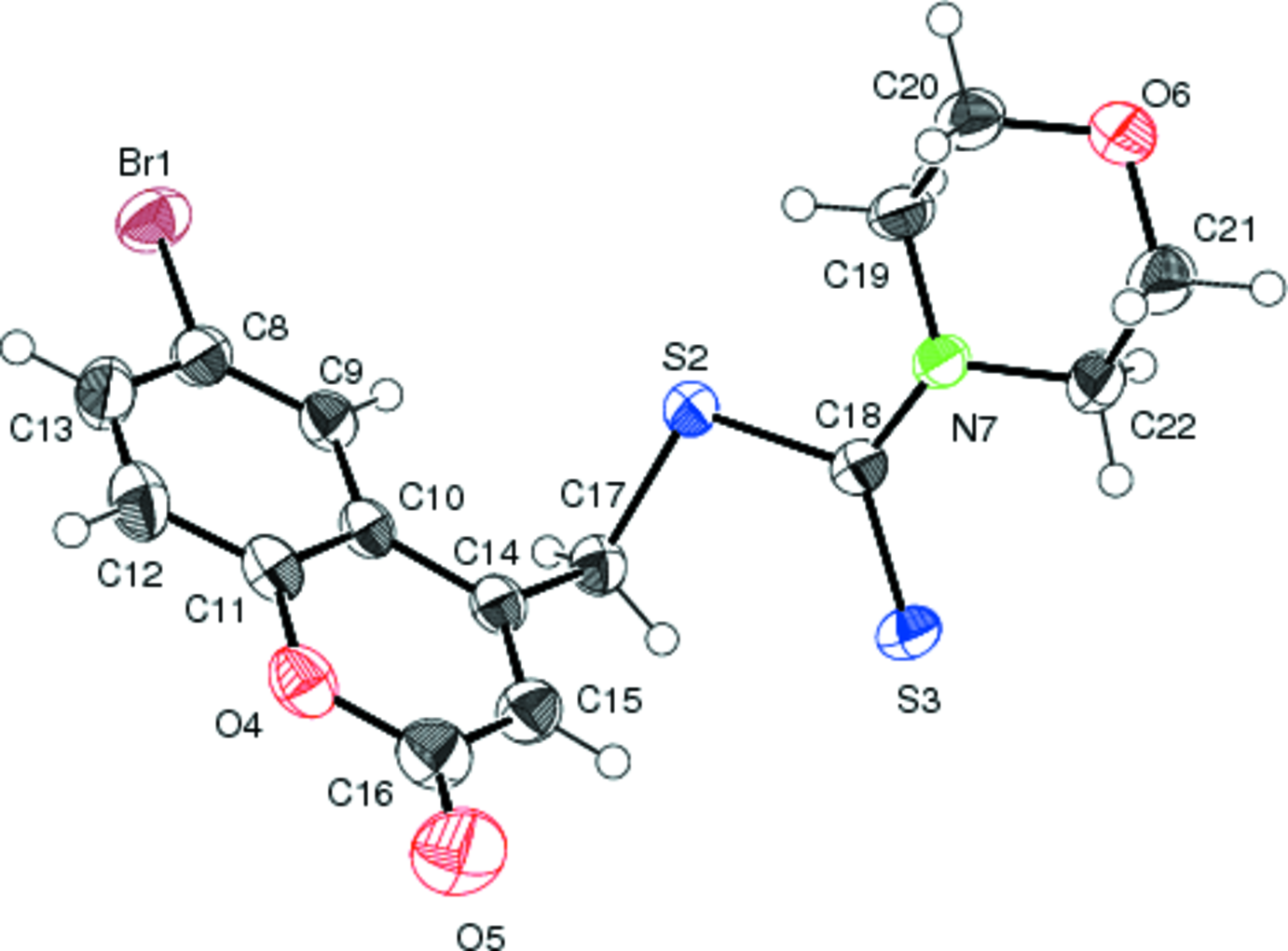

Supplement: Supplementary file 4 [file e-71-0o489-fig1.tif]

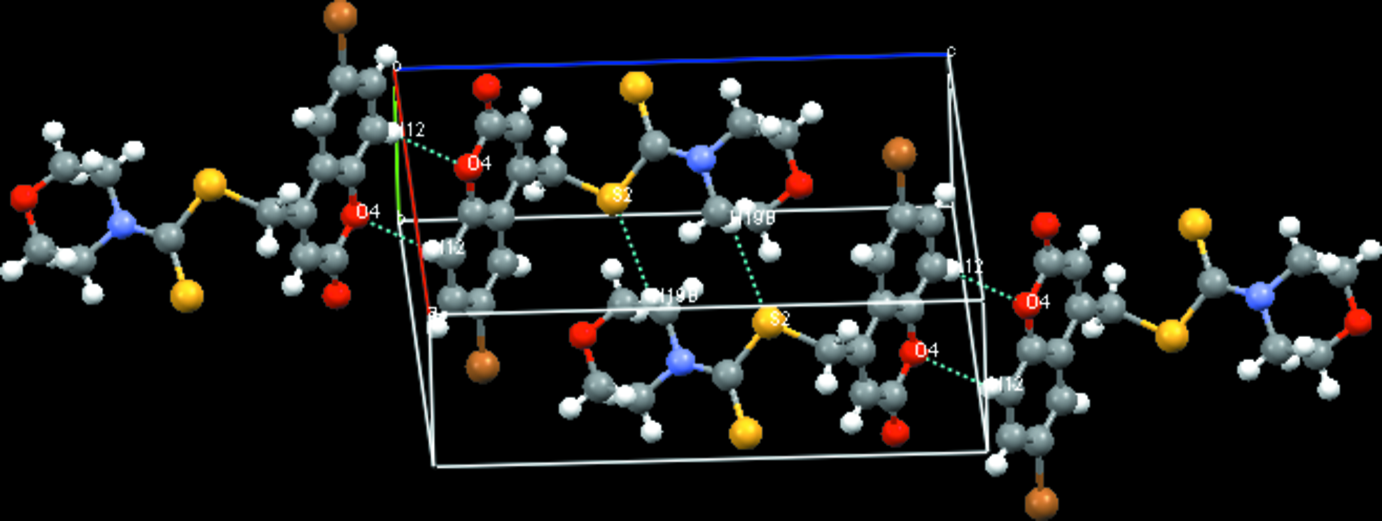

Supplement: Supplementary file 5 [file e-71-0o489-fig2.tif]
